# A novel CCR2 antagonist inhibits atherogenesis in apoE deficient mice by achieving high receptor occupancy

**DOI:** 10.1038/s41598-017-00104-z

**Published:** 2017-03-03

**Authors:** Ilze Bot, Natalia V. Ortiz Zacarías, Wilhelmus E. A. de Witte, Henk de Vries, Peter J. van Santbrink, Daniël van der Velden, Mara J. Kröner, Dirk-Jan van der Berg, Dean Stamos, Elizabeth C. M. de Lange, Johan Kuiper, Adriaan P. IJzerman, Laura H. Heitman

**Affiliations:** 1 Division of Biopharmaceutics, Leiden Academic Centre for Drug Research (LACDR), Leiden University, Leiden, the Netherlands; 2 Division of Medicinal Chemistry, Leiden Academic Centre for Drug Research (LACDR), Leiden University, Leiden, the Netherlands; 3 Division of Pharmacology, Leiden Academic Centre for Drug Research (LACDR), Leiden University, Leiden, the Netherlands; 4 Vertex Pharmaceuticals, San Diego, CA USA

## Abstract

CC Chemokine Receptor 2 (CCR2) and its endogenous ligand CCL2 are involved in a number of diseases, including atherosclerosis. Several CCR2 antagonists have been developed as potential therapeutic agents, however their *in vivo* clinical efficacy was limited. In this report, we aimed to determine whether 15a, an antagonist with a long residence time on the human CCR2, is effective in inhibiting the development of atherosclerosis in a mouse disease model. First, radioligand binding assays were performed to determine affinity and binding kinetics of 15a on murine CCR2. To assess the *in vivo* efficacy, western-type diet fed apoE^−/−^ mice were treated daily with 15a or vehicle as control. Treatment with 15a reduced the amount of circulating CCR2^+^ monocytes and the size of the atherosclerotic plaques in both the carotid artery and the aortic root. We then showed that the long pharmacokinetic half-life of 15a combined with the high drug concentrations ensured prolonged CCR2 occupancy. These data render 15a a promising compound for drug development and confirms high receptor occupancy as a key parameter when targeting chemokine receptors.

## Introduction

The chemokine system comprises more than 20 different chemokine receptors, which belong to the class A or rhodopsin-like family of G protein-coupled receptors (GPCRs). Almost 50 chemokine ligands play a critical role in the immune system, mediating the migration and differentiation of immune cells during homeostasis and inflammation^1^. Dysregulation of this system can lead to a variety of different pathologies, including inflammatory and autoimmune diseases^2, 3^. For instance, preclinical evidence suggests that CC Chemokine Receptor 2 (CCR2) and its high-affinity ligand CCL2 are involved in the pathogenesis of atherosclerosis^4–6^, neuropathic pain^7^ and multiple sclerosis^8^. Genetic knockout of CCR2 (CCR2^−/−^) in the Apolipoprotein E-deficient (apoE^−/−^) mouse model of atherosclerosis resulted in a significant decrease in lesion size compared to apoE^−/−^ controls, which was caused by a reduction in monocyte/macrophage recruitment to the atherosclerotic lesion^4–6^. Similar results have been reported in studies with genetic knockout of CCL2 in both the low density lipoprotein receptor-deficient (*LDLr* ^−/−^) and the human Apolipoprotein B transgenic mouse models^9, 10^. These findings suggest that the CCR2/CCL2 axis is critically involved in the mobilization and recruitment of monocytes to the early atherosclerotic plaque.

**Figure 1:**
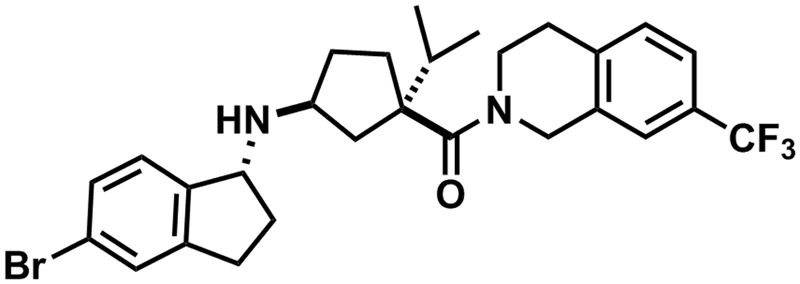
Chemical structure of CCR2 antagonist 15a.

The involvement of CCR2 in pathologies such as atherosclerosis has resulted in many efforts to develop biologic and small molecule antagonists targeting this receptor. However, despite inhibitory effects on for example monocyte recruitment^11^, a very limited number of compounds has shown *in vivo* efficacy in inhibiting atherosclerosis. In this regard, the characterization of the drug-target residence time (RT)—a measure of the ligand-receptor complex lifetime—has been proposed as a good predictor of *in vivo* efficacy and safety^12–15^. Although the link from *in vitro* kinetics to *in vivo* outcomes has only been studied in few drug targets, these studies confirm the importance of characterizing the RT of drug candidates^16–21^. Yet, the impact of RT to prolong the duration of effect can only be assessed when considering the whole target biology and pharmacokinetic properties of the drug of interest^22, 23^. Inclusion of RT in early hit-to-lead optimization has also been recently used for the development of high-affinity and long-RT CCR2 antagonists^24, 25^. The combination of structure-affinity and structure-kinetics optimization resulted in the discovery of compound 15a (Fig. 1), an orthosteric CCR2 small molecule antagonist with high affinity of 2.4 nM and a RT of 714 min for human CCR2 (hCCR2)^24^. With such a prolonged CCR2 inhibition, 15a emerges as a potential candidate to evaluate the *in vivo* effects of long RT.

In this study, we aimed to evaluate the binding kinetics and pharmacokinetics of 15a for murine CCR2 (mCCR2) and determine whether this CCR2 antagonist is effective in an apoE^−/−^ mouse model of atherosclerosis. Using a combination of *in vitro* radioligand binding assays and *in vivo* studies, we show that prolonged CCR2 antagonism with 15a, due to high target occupancy, is linked to a robust and significant inhibition of atherogenesis in mice. These results support the need of achieving more than 90% continuous inhibition when developing chemokine receptor antagonists^26^. This study also highlights the importance of *in vitro* characterization of drug candidates in all relevant species for *in vivo* pre-clinical studies, in order to improve the translational value of animal models, and thus to reduce the attrition in drug discovery programs.

## Results

### Characterization of [^3^H]INCB3344 and 15a in mouse CCR2 (mCCR2)

**Figure 2:**
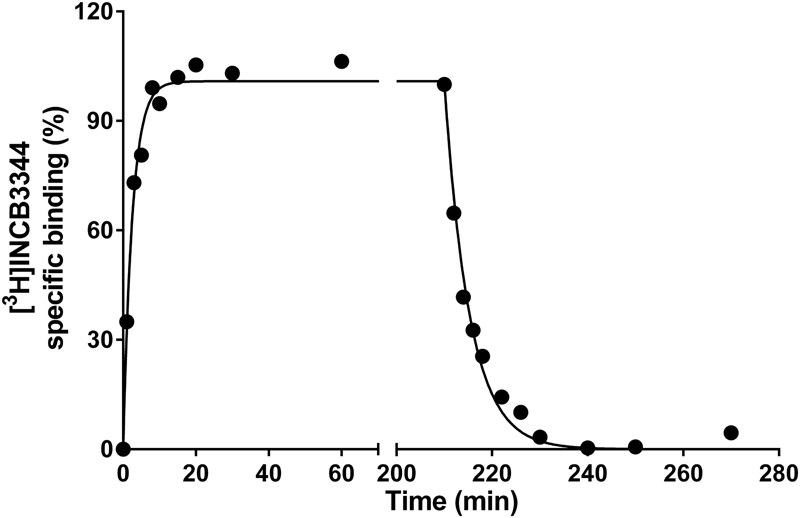
Kinetic characterization of [^3^H]INCB3344 in murine CCR2. Association and dissociation kinetics of 5 nM [^3^H]INCB3344 binding to membranes of CHO cell membranes transiently expressing murine CCR2 at 25 °C. Dissociation was initiated by the addition of 10 µM INCB3344. Association and dissociation data were fitted using a monophasic exponential association, or exponential decay model, respectively. Graphs shown are representative from one experiment performed in duplicate. Data for all association and dissociation experiments (n = 3) are presented in Table 1.

To determine the binding affinity and the kinetic profile of 15a at mCCR2, the radioligand [^3^H]INCB3344 was first characterized by performing association and dissociation binding assays in membranes of CHO cells transiently expressing mCCR2. At 25 °C, binding of [^3^H]INCB3344 to mCCR2 reached equilibrium around 20 min and was best fit with a one-phase exponential model that yielded an association (*k*_*on*_) rate of 0.030 nM^−1 ^min^−1^. Dissociation of the radioligand from mCCR2 was also best fit with a one-phase model that yielded a dissociation rate (*k*_*off*_) of 0.227 min^−1^ (Fig. 2 and Table 1). These values resulted in a kinetic dissociation binding constant (*K*_*D*_) of 7.6 nM and a RT of 4.4 min for this receptor (Table 1).

**Table 1:** Kinetic characterization of [^3^H]INCB3344 and 15a in membranes of CHO cells transiently expressing murine CCR2 (mCCR2).

	[^3^H]INCB3344 (hCCR2b^*^)	15a (hCCR2b^†^)
KRI^‡^	1.0 (1.0)	1.6 (1.7)
*k*_*on*_ (nM^−1 ^min^−1^)	0.030 ± 0.003 (0.054)	0.007 ± 0.001 (0.008)
*k*_*off*_ (min^−1^)	0.227 ± 0.031 (0.013)	0.051 ± 0.008 (0.0014)
*K*_*D*_^§^ (nM)	7.6 ± 1.3 (0.23)	7.4 ± 1.5 (0.175)
RT^||^ (min)	4.4 ± 0.6 (76)	19.7 ± 3.1 (714)

Values are means ± S.E.M of three separate experiments performed in duplicate.

^*^Values of [^3^H]INCB3344 for hCCR2b were taken from Vilums *et al.* ^25^.

^†^Values of 15a for hCCR2b were taken from Vilums *et al.* ^24^.

^‡^Kinetic rate index (KRI) = B_t1_/B_t2_ (see Materials and Methods).

^§^ *K*_*D*_ = *k*_*off*_/*k*_*on*_.

^||^RT = 1/*k*_*off*_.

**Figure 3:**
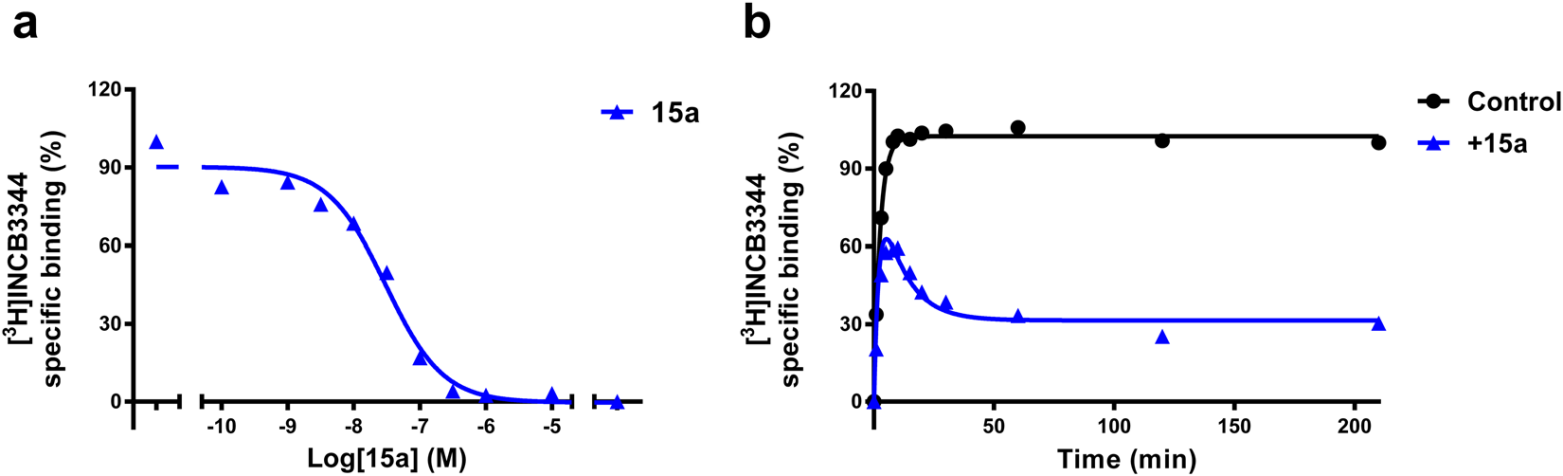
Characterization of 15a in murine CCR2. (**a**) Displacement of [^3^H]INCB3344 binding from CHO cell membranes transiently expressing murine CCR2 at 25 °C, upon addition of increasing concentrations of compound 15a. (**b**) Competition association of 5 nM [^3^H]INCB3344 binding to membranes of CHO cells transiently expressing murine CCR2 at 25 °C, in the absence or presence of 30 nM 15a. Data were fitted to the competition association model of Motulsky and Mahan^27^. Graphs shown are representative from one experiment performed in duplicate. Data for kinetic experiments (n = 3) are presented in Table 1.

After the characterization of [^3^H]INCB3344, we performed displacement experiments at 25 °C with 15a to determine its affinity for mCCR2. 15a was able to displace [^3^H]INCB3344 binding in a concentration-dependent way, with a K_i_ value of 10.6 nM (Fig. 3a). In addition, a [^3^H]INCB3344 competition association assay was performed in order to determine the kinetic profile of unlabeled 15a. Figure 3b shows a representative competition association curve of [^3^H]INCB3344 to mouse CCR2, in the absence or presence of 30 nM 15a (approximately 3-fold its K_i_ value). We used 30 nM 15a as this concentration provided a sufficient window for data analysis. Association curves of [^3^H]INCB3344 in the presence of 15a in mCCR2 resulted in the typical overshoot characteristic for long RT compounds (Fig. 3b) and a KRI > 1.0 (KRI = 1.6). By fitting the curves to the competition association model of Motulsky and Mahan^27^, it was possible to determine the *k*_*on*_ and *k*_*off*_ values of 15a: 0.007 ± 0.001 nM^−1 ^min^−1^ and 0.051 ± 0.008 min^−1^, respectively. These results allowed the calculation of the kinetic *K*_*D*_ (7.4 ± 1.5 nM), which was comparable to the obtained K_i_ value, and confirmed a slower dissociation—and a longer RT of 20 min—compared to [^3^H]INCB3344 (RT = 4.4 min, Table 1). For comparison, the kinetic parameters of 15a and [^3^H]INCB3344 on human CCR2 (hCCR2) are also shown in Table 1, as previously published by Vilums *et al*.^24, 25^.

### 15a reduces circulating CCR2^+^ monocyte levels

**Figure 4:**
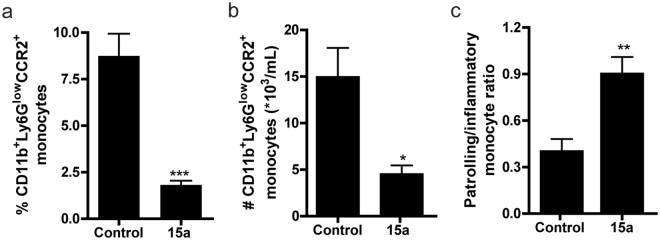
Effect of 15a treatment on the amount of monocytes. (**a**) At 2 hours after injection and two weeks of treatment, 15a reduced the relative amount of circulating CCR2^+^ monocytes (defined as CD11b^+^Ly6G^low^ cells). (**b**) At 18 hours after the last injection (after 4 weeks of treatment), the number of circulating CD11b^+^Ly6G^low^CCR2^+^ cells was significantly reduced by 15a treatment. (**c**) The ratio of patrolling monocytes and pro-inflammatory monocytes was significantly increased upon treatment with 15a (*P < 0.05, **P < 0.01, ***P < 0.001). Graphs shown are representative from one experiment with n = 5 per group in panel (a) and n = 6 per group in panels (b) and (c).

In this study, apoE^−/−^ mice were treated with the orthosteric antagonist 15a or vehicle control for four weeks. During the study, body weight, body weight gain and plasma total cholesterol levels were not affected by administration of the CCR2 antagonist (Supplementary Fig. S1). Liver morphology was similar between the controls and the 15a treated mice, and 15a did not affect liver mRNA expression of the toxicity markers ALT, apoH and GC. mRNA levels of AST were even somewhat decreased in the 15a treated group (P = 0.02, Supplementary Fig. S2). Interestingly, treatment with 15a resulted in increased plasma CCL2 levels, while plasma IL-6 levels remained unaffected (Supplementary Fig. S3). After two weeks of treatment, we determined CCR2^+^ monocyte levels (defined as CD11b^+^Ly6G^low^ cells) in blood of control and 15a treated mice at two hours after injection by means of flow cytometry. At that time point, we observed a significant reduction in the percentage of circulating CCR2^+^ monocytes in the 15a treated mice (Fig. 4a, controls: 8.7 ± 1.2%, 15a: 1.8 ± 0.2%, P = 0.0004).

At the time of sacrifice, the total number of monocytes was significantly reduced in the mice treated with 15a, while total lymphocyte and neutrophil numbers remained unaffected (Supplementary Fig. S4). Flow cytometry analysis further confirmed that 15a did not affect the number of neutrophils, CD19^+^ B cells, CD4^+^ or CD8^+^ T cells in blood or spleen, nor did we detect any effects of the antagonist on splenic or circulating Tregs (data not shown). Of note, these analyses were performed at 18 hour after the last injection of the antagonist. At that time-point, we still observed that the number of circulating CCR2^+^ monocytes was significantly reduced in the 15a treated mice compared to controls (Fig. 4b, controls: 14.9 ± 3.2*10^3^ cells/mL versus 15a: 4.5 ± 1.0*10^3^ cells/mL, P = 0.01). The ratio of patrolling CD11b^+^Ly6C^low^CX3CR1^+^ monocytes versus inflammatory CD11b^+^Ly6C^high^CCR2^+^ monocytes in blood increased upon treatment with 15a (Fig. 4c), which was not only due to a reduction in the number of CCR2^+^ monocytes, but also caused by a significant 1.5-fold increase in the amount of CD11b^+^Ly6C^low^CX3CR1^+^ monocytes. We did not observe any change in the percentage of circulating CCR5+ monocytes in both the treated and untreated mice, which suggests a selective interaction of 15a with CCR2 (Supplementary Fig. S5).

### CCR2 antagonism inhibits atherosclerotic plaque development

**Figure 5:**
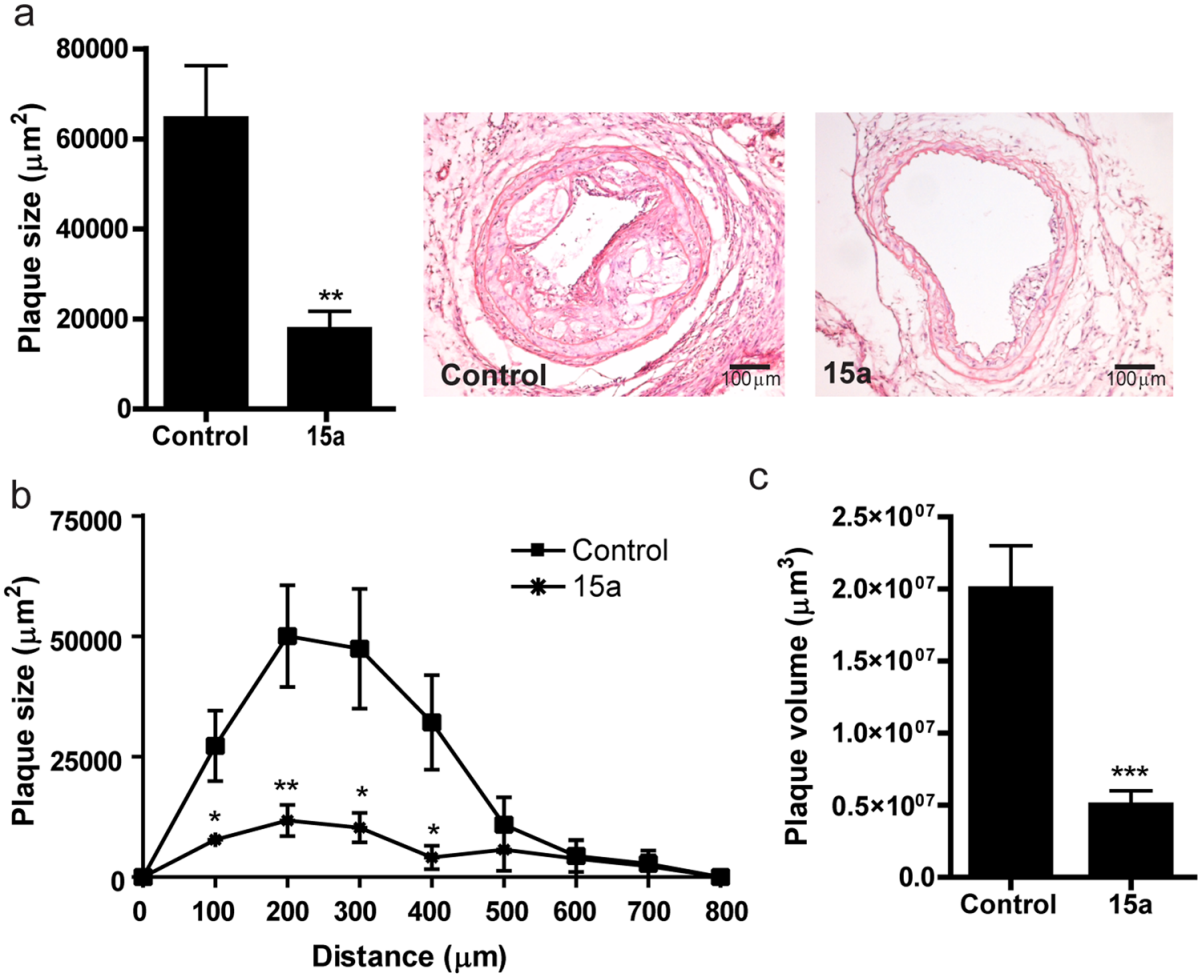
Inhibition of lesion development in the carotid artery by 15a. (**a**) The CCR2 antagonist 15a significantly reduced atherosclerotic plaque size in the carotid artery at the site of maximal stenosis. (**b**) Similarly, plaque volume, given as plaque size at increasing distance from the collar and (**c**) as total volume in µm^3^ was significantly reduced by 15a. Micrographs show representative images of the individual groups (100X magnification). *P < 0.05, **P < 0.01, ***P < 0.001. Graphs shown are representative from one experiment with n = 10 controls versus n = 9 15a-treated mice.

Atherosclerotic plaque size in the carotid artery at the site of maximal stenosis was reduced from 64.4 ± 11.8*10^3 ^µm^2^ in control mice to 17.6 ± 4.1*10^3 ^µm^2^ in mice treated with 15a (Fig. 5a, −73%, P = 0.002). Furthermore, as depicted in Fig. 5b,c, plaque volume throughout the carotid arteries was reduced from 1.75 ± 0.33*10^7 ^µm^3^ in control mice to 0.46 ± 0.13*10^7 ^µm^3^ in 15a treated mice (P = 0.003). In addition, relative necrotic area was reduced from 35.3 ± 8.2% in controls to 7.6 ± 4.5% in the 15a treated mice (P = 0.01).

**Figure 6:**
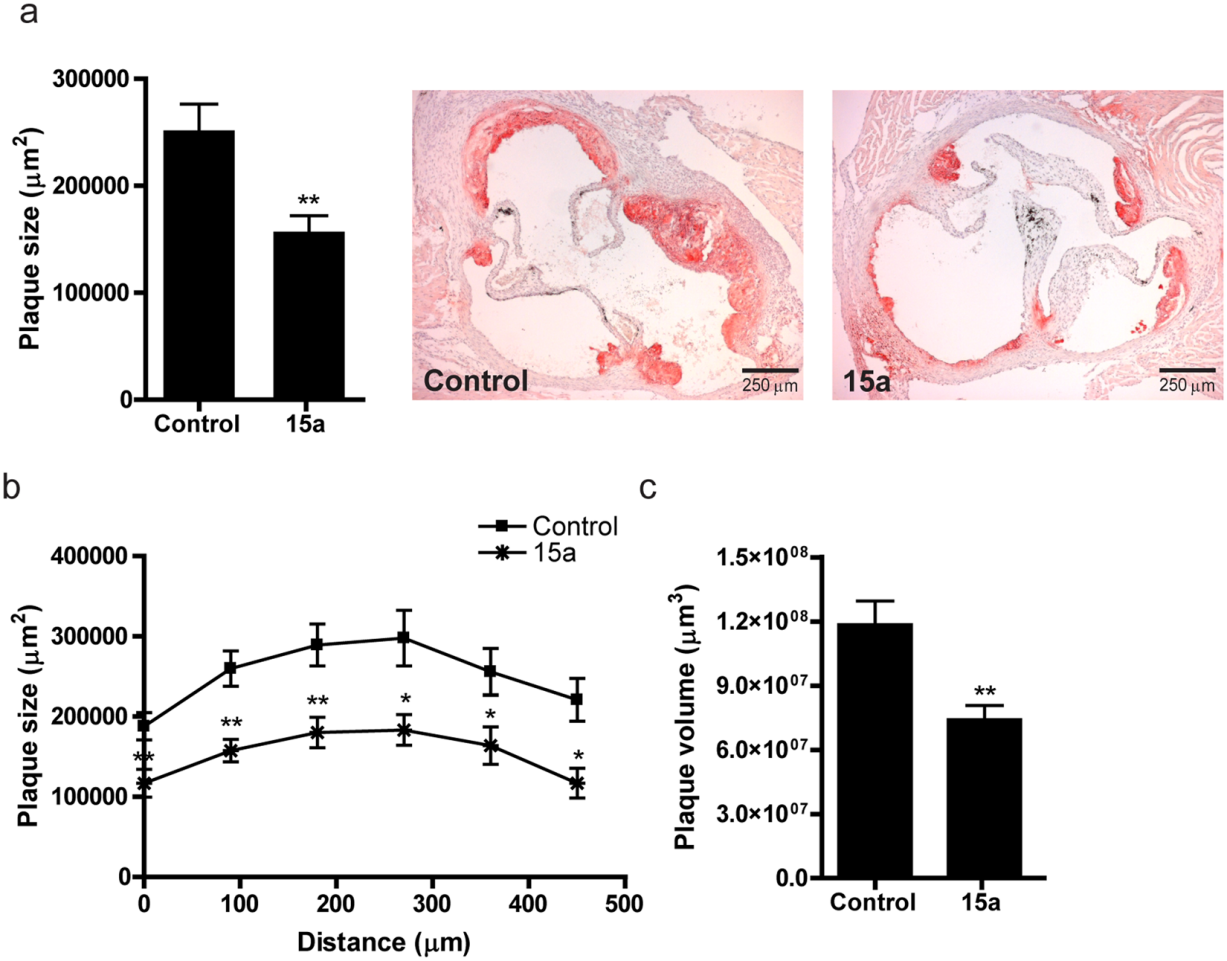
Inhibition of lesion development in the aortic root by 15a. (**a**) Also in the aortic root, 15a inhibited atherosclerotic lesion development after 6 weeks of Western type diet. (**b**) Lesion area in the aortic roots presented for each group every 90 μm of the aortic root. (**c**) Plaque volume was also significantly reduced in the 15a-treated group. *P < 0.05, **P < 0.01. Graphs shown are representative from one experiment with n = 10 controls versus n = 9 15a-treated mice.

In the aortic root, average plaque size was significantly reduced by treatment with 15a (Fig. 6a, controls: 215.9 ± 24.6*10^3 ^µm^2^, 15a: 157.0 ± 15.7*10^3 ^µm^2^, P = 0.005) and also aortic root plaque volume was lower in the 15a treated mice (Fig. 6b,c, controls: 11.8 ± 1.2*10^7 ^µm^3^, 15a: 7.4 ± 0.7*10^7^ µm^3^, P = 0.005).

**Figure 7:**
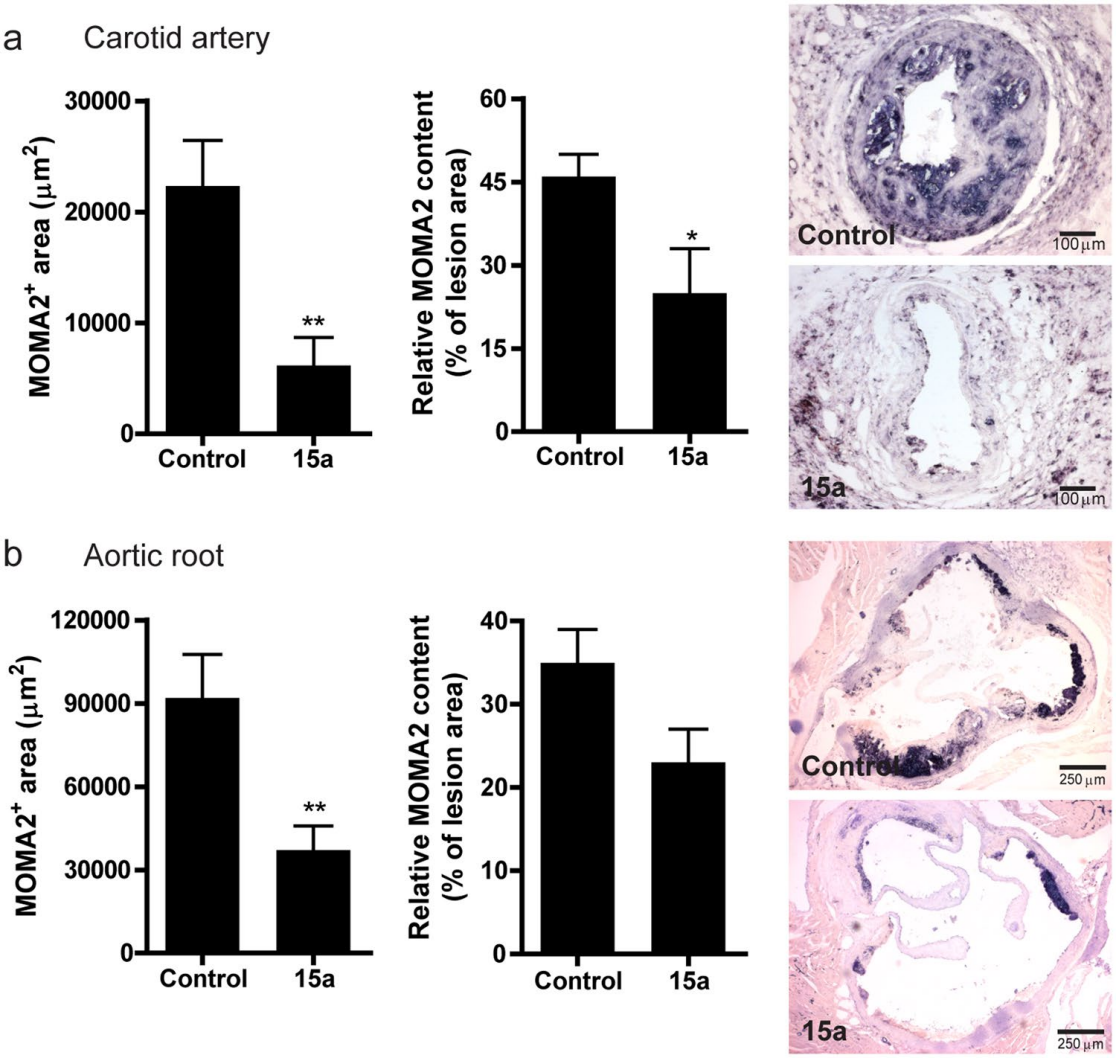
Macrophage content in carotid artery and aortic root atherosclerotic lesions. (**a**) Macrophage content in the carotid artery lesions as measured by MOMA2 staining was reduced upon treatment with 15a, both in total macrophage positive area (left panel) and relatively as percentage of lesion size (right panel). Micrographs show representative images of the individual groups (100X magnification). (**b**) Also in the aortic root, 15a significantly reduced the macrophage^+^ area (left panel). Relative macrophage content did not significantly differ between the groups (right panel). Micrographs show representative images of the individual groups (40X magnification). *P < 0.05, **P < 0.01. Graphs shown are representative from one experiment with n = 10 controls versus n = 9 15a-treated mice.

The macrophage content as measured by MOMA2 staining revealed that the macrophage positive area of the carotid artery plaques was significantly reduced in the 15a treated mice (Fig. 7a, left panel; controls: 22.3 ± 4.1*10^3^ µm^2^, 15a: 6.2 ± 2.5*10^3^ µm^2^, P = 0.005). Similarly, relative macrophage content (corrected for lesion size) was reduced in the 15a treated group (Fig. 7a, right panel; controls: 46 ± 4%, 15a: 25 ± 8%, P = 0.02). In the aortic root, macrophage positive area was also significantly lower in the 15a treated mice (Fig. 7b, controls: 92.0 ± 15.6*10^3 ^µm^2^, 15a: 37.2 ± 8.7*10^3 ^µm^2^, P = 0.008).

### Pharmacokinetics-based prediction of target occupancy

**Figure 8:**
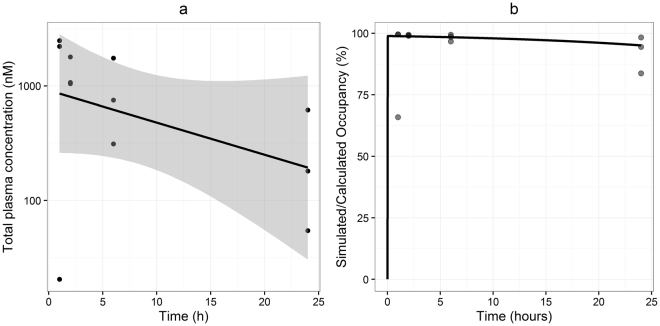
Total plasma concentrations, calculated and simulated target occupancy. (**a**) Observed plasma concentrations (dots), linear fit of the data (line) and 95% confidence interval of the linear regression (shaded area). (**b**) Equilibrium occupancy derived from the experimentally observed plasma concentrations (dots) and simulated occupancy on basis of the observed binding kinetics and plasma elimination rate (line). Graphs shown are representative from one experiment (n = 3 per time point).

To investigate the role of pharmacokinetics in the generation of the effective drug response of 15a, we measured the plasma levels of 15a over a 24 hour period using a LC-MS method. Following a single intraperitoneal dose of 5 mg/kg, plasma levels of 15a varied from 12 to 1456 ng/mL 1 h after administration; 2 h after dosing, plasma levels were above 600 ng/mL in all individual mice (613 to 1048 ng/mL), which remained relatively constant at 6 h after administration (182 to 1024 ng/mL) and fell to 32 to 331 ng/mL at 24 h after administration (Fig. 8a). Pharmacokinetic analysis at this dose revealed a relatively slow pharmacokinetic profile (Fig. 8a, elimination half-life = 10.7 hours), with an elimination half-life that exceeds by far the determined RT in mCCR2. Using these plasma concentrations, we predicted the target occupancy attained by 15a using two different approaches: i) calculation of “equilibrium” target occupancy using equation (1) as shown in Materials and Methods, which accounts for both the pharmacokinetics and affinity of 15a, and ii) simulation of target occupancy using equations (3–5), which account for the pharmacokinetics and binding kinetics of this compound. These calculations demonstrated that the combination of slow pharmacokinetics (Fig. 8a) and high drug concentrations relative to affinity leads to a high target occupancy of >90% for 24 hours (Fig. 8b, dots). This was confirmed by the simulations of receptor occupancy (Fig. 8b, line), indicating that a simpler calculation method (equation (1)) is sufficient to predict receptor occupancy in this study. Comparing the long duration of target occupancy for equilibrium binding with the RT of 15a (of 24 minutes) makes clear that RT is not a contributing factor in prolonging target occupancy under these conditions. In this regard, equation (2) allowed us to determine the maximal value of *k*_*off*_ that could contribute to a prolonged duration of target occupancy. As previously demonstrated, the level of target saturation can be used to compare the plasma elimination with the *k*_*off*_, by multiplying the pharmacokinetic elimination rate with a factor (1-BF), where BF represents the percentage of target occupancy divided by 100^28^. This means that if the target occupancy needs to be above 90% for effective treatment, the *k*_*off*_ of the drug candidate needs to be ten times lower than the pharmacokinetic elimination rate.

## Discussion and Conclusions

In this study, we investigated the efficacy of the novel CCR2 antagonist 15a—designed to display a long residence time (RT) on human CCR2 (hCCR2)—in inhibiting the development of atherosclerosis in apoE^−/−^ mice. Characterization of the drug-target complex lifetime or RT in early phases of the drug discovery and development process might help to improve the prediction of *in vivo* efficacy and safety^12–15^. Evidence from retrospective studies has shown that several marketed drugs exhibit a long RT on their target^13, 29^, prompting the prospective optimization of both affinity and kinetic properties of compounds for a variety of targets^17, 25, 30–32^. In this regard, 15a was discovered by optimizing a reported lead in the development of MK-0812, a Merck CCR2 antagonist that has failed in clinical trials due to lack of efficacy^33^. Although the RT of MK-0812 in mCCR2 is not known, in hCCR2 it exhibited an almost 8-fold shorter RT than compound 15a (92 min versus 714 min, respectively)^24^. Thus, our selection of 15a was based on the need of advancing drug candidates with high affinity and favorable binding kinetics. In this study, we first determined the kinetic profile of 15a on mouse CCR2 (mCCR2), as the pharmacological properties of drug candidates, including the kinetic profile, can differ between species orthologues^34^. This renders it necessary to characterize the binding kinetics of lead compounds in all relevant species for preclinical testing^12^. Using [^3^H]INCB3344 we were able to determine the binding affinity and the kinetic profile of 15a in mCCR2. The determination of RT using the competition association model of Motulsky and Mahan^27^ confirmed the longer RT of 15a in comparison with the control [^3^H]INCB3344, a CCR2 receptor antagonist with similar affinity for mCCR2. This is in line with previous studies in hCCR2, where 15a showed a RT of 714 min compared with 76 min of [^3^H]INCB3344 (Table 1)^24^. However, a clear difference was found between species, as 15a dissociates much faster from mCCR2 than from hCCR2: 15a has a lifetime of less than 30 min on mCCR2, but more than 10 hours on hCCR2. This poor translation of kinetic parameters between species represents a clear limitation in understanding the effect of RT in *in vivo* efficacy, adding an extra level of complexity when selecting relevant animal models for preclinical studies.

Next we showed that 15a significantly reduced the number of circulating inflammatory CCR2^+^ monocytes and that 15a was very effective in inhibiting atherosclerotic plaque development in both the carotid artery and the aortic root of apoE^−/−^ mice. In addition, relative macrophage content in the lesion was reduced in the 15a treated mice at both sites of lesion development. CCR2 was previously shown to be predominantly involved in lesion initiation^4, 6^, but seemed less effective in inhibiting lesion progression. For therapeutic application, halting lesion progression would be favorable to prevent an existing plaque to progress to an advanced and unstable lesion. In this study, 15a almost completely blocked lesion initiation in the carotid artery, as treatment was commenced immediately from time of collar placement and thus of lesion development at that site. In the aortic root, early atherosclerosis had already developed before treatment was initiated, and treatment was also effective in inhibiting plaque progression in this location. Aiello *et al*. showed that treatment with the CCR2 antagonist INCB3344 failed to decrease the size of early and advanced atherosclerotic lesions in apoE^−/−^ mice^11^, which may be related to both the shorter RT of this drug in mCCR2, and the use of an ineffective dose. Although the plasma levels of INCB3344 were not investigated in this study, previous data suggest that a single oral dose of 50 mg/kg leads to approximately 75% CCR2 inhibition in mice^35^. In the study by Aiello *et al*., inflammatory monocyte levels were significantly reduced up to 6 hours after oral infusion, which was lost at 9 hours, while we still observed a >60% reduction in circulating CCR2^+^ monocyte numbers at 18 hours after treatment, suggestive of a longer therapeutic effect. Interestingly, we also observed an increase in the relative amount of Ly6C^low^CX3CR1^+^ monocytes, which is indicative of a more patrolling response^36^. Similarly as in the study by Aiello *et al*., we did not observe any effects on bone marrow monocyte subpopulations upon treatment (data not shown), suggesting that the reduction observed on circulating CCR2^+^ monocytes is not due to changes in migration of these cells from the bone marrow. Further studies should shed more light on the exact mechanisms of depletion of this specific monocyte subset from the circulation, and how this affects the immune system, including effects on other chemokine receptors expressed by monocytes. In both studies however, plasma cholesterol levels were not affected, while an increase in plasma CCL2, one of the endogenous ligands for CCR2, was observed, which may be explained by the fact that clearance of CCL2 is mediated by CCR2^37^. Noteworthy, treatment with 15a resulted in more than 70% reduction in carotid artery lesion size, which even exceeds the effects of the CCR2/CCR5/CXCR3 triple antagonist TAK-779 in inhibiting atherogenesis^38^, suggesting that inhibition of a single receptor, CCR2, might be sufficient for the therapeutic effect in atherosclerosis.

Finally we also investigated the pharmacokinetics of 15a in apoE^−/−^ mice, which allowed us to determine drug exposure and predict the target occupancy achieved by 15a after a single dose. For a drug to exert its pharmacological effect, it must be bound to its target, making target occupancy a requisite for any *in vivo* effect. Previous analysis of clinical trials with CCR1 antagonists led to the conclusion that a receptor occupancy of >90% is required for an effective anti-inflammatory response^26, 39^. Calculation of CCR2 occupancy levels in our study revealed that, after a single dose, 15a was able to block more than 90% of the receptors for over 24 hours. Such high level of target engagement can be achieved by slow drug-target dissociation or by target-saturating drug concentrations. In our case, the calculated high receptor occupancy is the result of saturating concentrations and slow plasma elimination of 15a. For RT to play a key role in increasing the target occupancy, the *k*_*off*_ of the drug candidate must at least surpass its pharmacokinetic elimination rate^22, 23^. However, the direct comparison of the pharmacokinetic elimination rate and the *k*_*off*_ is only informative when the target occupancy is low, since it assumes that the declines of the target occupancy and the unbound drug concentration are parallel lines on a semi-logarithmic scale. For high levels of target occupancy (i.e. high target saturation), a steep decline of unbound drug concentrations is not directly reflected in a steep decline of target occupancy^28^, as we have predicted. This reasoning was used to determine the *k*_*off*_ value required to prolong target occupancy even further: at least a ten-fold difference between the dissociation rate and the plasma elimination rate is required. Although the contribution of target saturation is not always recognized in literature^19^, we provide evidence that the use of saturating concentrations can result in the required occupancy levels for an anti-inflammatory effect. However, caution must be taken in order to avoid safety risks and toxicity with high doses. These results highlight the need of obtaining pharmacokinetic data and using experimental or mathematical approaches to determine receptor occupancy in early preclinical studies. In this regard, we have established that a simple “equilibrium” occupancy calculation can be sufficient to explain the *in vivo* efficacy in this study, providing a simple method that can be readily used in a range of *in vitro* and *in vivo* preclinical studies. This calculation method allows for further optimization towards a clinical candidate, for example by developing an oral dosing regimen to improve patient compliance.

Although the role of the CCR2:CCL2 axis in the development of atherosclerosis has been recognized in literature, previous studies with CCR2 small-molecule antagonists failed to successfully inhibit atherosclerosis^11, 40^. We are the first to prove that CCR2 antagonism with a selective small molecule antagonist can result in effective atherogenesis inhibition. The results of our *in vivo* study provide direct evidence that high receptor occupancy might be one of the key factors to improve the translation from *in vitro* findings into *in vivo* efficacy in inflammatory diseases. In addition, our findings in the *in vitro* study support the need of characterizing the affinity and kinetic profile of drug candidates in multiple species, as a way to improve preclinical and clinical translation of efficacy and safety findings^12, 41^. To conclude, the CCR2 antagonist 15a emerges as a potential candidate for further drug development to inhibit atherosclerotic lesion development and progression, and may also be of therapeutic value for other diseases involving CCR2.

## Materials and Methods

### Chemicals and reagents

CCR2 antagonists INCB3344, 15a ((1*S*,3*R*)-3-(((*R*)-5-bromo-2,3-dihydro-1*H*-inden-1-yl)amino)-1-isopropylcyclopentyl)(7-(trifluoromethyl)-3,4-dihydroisoquinolin-2(1*H*)-yl)methanone) and the internal standard 15b ((1*S*,3*R*)-3-(((*S*)-5-bromo-2,3-dihydro-1*H*-inden-1-yl)amino)-1-isopropylcyclopentyl)(7-(trifluoromethyl)-3,4-dihydroisoquinolin-2(1*H*)-yl)methanone) were synthesized in-house as described previously^24, 42, 43^. [^3^H]INCB3344 (specific activity 32 Ci mmol^−1^) was custom-labeled by Vitrax (Placentia, CA) as a racemic mixture of the two isomers *N*-(2-(((3*S*,4*S*)-1-((1*r*,4*S*)-4-(benzo[d][1,3]dioxol-5-yl)-4-hydroxycyclohexyl)-4-ethoxypyrrolidin-3-yl)amino)-2-oxoethyl)-3-(trifluoromethyl)benzamide and *N*-(2-(((3*R*,4*R*)-1-((1*r*,4*R*)-4-(benzo[d][1,3]dioxol-5-yl)-4-hydroxycyclohexyl)-4-ethoxypyrrolidin-3-yl)amino)-2-oxoethyl)-3-(trifluoromethyl)benzamide. Bovine serum albumin (BSA, fraction V) was purchased from Sigma (St. Louis, MO, USA). Bicinchoninic acid (BCA) and BCA protein assay reagent were obtained from Pierce Chemical Company (Rockford, IL, USA). Acetonitrile (LC-MS grade), trifluoroacetic acid (TFA) and ULC-MS water were from Biosolve (Valkenswaard, the Netherlands); and ethyl acetate from Baker (Deventer, The Netherlands). pcDNA3.1+ plasmid containing the murine CCR2 (mCCR2) with an hemagglutinin (HA) epitope tag at the N-terminus was cloned in-house. All other chemicals were obtained from standard commercial sources.

### Cell culture

Chinese hamster ovary (CHO) cells were cultured at 37 °C and 5% CO_2_ in Dulbecco’s Modified Eagle Medium: Nutrient Mixture F-12 (DMEM/F-12) supplemented with 10% (v/v) newborn calf serum, 50 IU/mL penicillin and 50µg/mL streptomycin. Cells were subcultured twice a week on 10-cm ø plates, at a ratio of 1:20 to 1:40 using trypsinization.

### Transfections

Transient transfection of CHO cells with HA-tagged mouse CCR2 (mCCR2) was performed using a polyethyleneimine (PEI) method, as described previously^44^. Briefly, CHO cells were seeded at 50–60% confluence on 15-cm ø plates, transfected with 10 µg of plasmid DNA per plate, and incubated for 48 hrs at 37 °C and 5% CO_2_. Before transfection, the plasmid DNA was diluted in sterile 150 mM NaCl solution and mixed with PEI solution (1 mg/mL) to obtain a DNA:PEI mass ratio of 1:6. Finally, the culture medium of the cells was refreshed and 1 mL of DNA/PEI mixture was added to the cells, after incubation of the mixture for 20 min at room temperature.

### Cell membrane preparation

Membranes were prepared as described before^44^. Briefly, cells were detached from 15-cm ø plates by scraping them with 5 ml phosphate-buffered saline (PBS), and the membranes were separated from the cytosolic fractions by several centrifugation and homogenization steps. The remaining membrane pellet was resuspended in ice-cold Tris buffer (50mM Tris-HCl, pH 7.4) containing 5 mM MgCl_2_. Membrane protein concentrations were measured using a bicinchoninic acid (BCA) protein determination with bovine serum albumin (BSA) as a standard^45^.

### [^3^H]INCB3344 Binding assays

Radioligand binding assays were performed in a 100 µL reaction volume containing 50 mM Tris-HCl buffer (pH 7.4), 5 mM MgCl_2_, 0.1% 3-[(3-cholamidopropyl)-dimethylammonio]-1-propanesulfonic acid (CHAPS), 5 nM [^3^H]INCB3344 and 50 to 60 µg of membrane protein at 25 °C. Non-specific binding was determined with 10 μM of the cold ligand INCB3344. Displacement assays were carried out using 9 concentrations of competing ligand, ranging from 0.1 nM to 100 μM, for 120 min of incubation. The kinetic parameters of 15a were also determined at 25 °C using a competition association assay as previously described^25, 27^. For association and competition association experiments, CHO-mCCR2 membranes were added to the reaction volume at different time points, in the absence or presence of 30 nM of competing ligand. For dissociation experiments, CHO-mCCR2 membranes were first incubated with 5 nM [^3^H]INCB3344 for 60 min. Dissociation was initiated by addition of 10 µM of INCB3344 at different time points. For all experiments, incubation was terminated by dilution with ice-cold 50 mM Tris-HCl buffer supplemented with 5 mM MgCl_2_ and 0.05% CHAPS. Separation of bound from free radioligand was performed by rapid filtration over a 96-well GF/B filter plate using a Perkin Elmer Filtermate-harvester (Perkin Elmer, Groningen, the Netherlands) in the case of displacement assays; or over GF/B filters using a Brandel harvester 24 (Brandel, Gaithersburg, MD, USA) for kinetic assays. Filter-bound radioactivity was determined by scintillation spectrometry after addition of Microscint scintillation cocktail (Perkin-Elmer, Groningen, the Netherlands). The P-E 2450 Microbeta2 plate counter (Perkin Elmer, Groningen, The Netherlands) was used for displacement assays, and the TRI Carb 2900 TR counter (Perkin Elmer, Groningen, The Netherlands) for kinetic assays. In all cases, total radioligand binding did not exceed 10% of the amount of radioligand added to prevent ligand depletion.

### Animals

This study was performed in compliance with Dutch government guidelines and the Directive 2010/63/EU of the European Parliament. All animal experiments were approved by the animal welfare committee of Leiden University (approval reference number 13213).

Male apoE^−/−^ mice (10–12 weeks old), obtained from the local animal breeding facility (Gorlaeus Laboratories, Leiden, The Netherlands), were fed a Western type diet, containing 0.25% cholesterol and 15% cocoabutter (SDS, Sussex, UK).

To measure plasma concentrations of 15a over time, Western type diet fed mice were injected with 5 mg/kg 15a, after which blood samples were taken at fixed time points (1, 2, 6 and 24 hours after injection). Blood samples were processed as described below under “Measurement of 15a plasma levels”. The final dose of 5 mg/kg/day was based on a pilot study in which two different doses (1.5 and 5 mg/kg/day) were tested using leucocyte migration towards the peritoneal cavity as a read-out. Results from this pilot study showed that 1.5 mg/kg per day was not effective in inhibiting migration, while 5 mg/kg per day was (data not shown). In addition, the dose of 5 mg/kg was also based on a previous study with TAK-779^38^, in which a dose of 5 mg/kg was used.

To determine the effect of the CCR2 antagonist on atherosclerotic lesion development, mice were fed a Western type diet, containing 0.25% cholesterol and 15% cocoabutter (SDS, Sussex, UK) throughout the experiment starting two week before surgery. Carotid artery plaque formation was induced by perivascular collar placement as described previously^46^ and from the time of surgery, the mice received daily intraperitoneal injections containing 15a (5 mg/kg/day) or vehicle control (n = 9–10 per group). During the experiment, total body weight and weight gain were measured. Serum total cholesterol levels were determined using an enzymatic colorimetric assay according to manufacturer’s protocol (Roche Diagnostics). Precipath (standardized serum; Boehringer Mannheim, Germany) was used as an internal standard. Plasma cytokine levels were measured by ELISA according to manufacturer’s protocol (BD Biosciences). Blood was collected weekly via tail cut. Total cell count and cellular differentiation patterns in blood were analyzed using an automated XT-2000iV veterinary hematology analyzer (Sysmex Europe GMBH, Norderstedt, Germany). After erythrocyte lysis, blood leukocyte suspensions were stained for surface markers, after which their expression was determined by FACS analysis (FACS Canto, BD Biosciences). After 4 weeks of lesion development, the animals were anaesthetized and blood was collected. After *in situ* perfusion through the left cardiac chamber, the carotid arteries and hearts were harvested.

### Measurement of 15a plasma levels

Blood plasma from mice was analyzed by LC-MS to determine 15a plasma levels. 20 μL of internal standard (compound 15b, a diastereomer of 15a^24^) and 20 μL of solvent C (20% (v/v) Acetonitrile and 0.1% (v/v) TFA in LC-MS water) were first mixed with 20 µL of plasma. A calibration curve from 2 to 200 ng/ml of 15a was prepared using solvent C. Calibration samples were prepared as normal samples using blanc plasma and calibration solution instead of solvent C. Aliquots of 40 µL of 1% TFA were added to the samples and mixed well. After addition of 900 µL of ethyl acetate, mixing was performed for 5 minutes at a titramax plate shaker (Heidolph, Swabach, Germany). Samples were then centrifuged at 15,000 × *g* during 10 minutes and 800 µL of the supernatant was evaporated in a vacuum centrifuge (Labconco, Kansas City, Missouri). The sample was reconstituted by mixing with 10 µL of acetonitrile, followed by addition of 40 µL solvent C. After a second centrifugation at 15,000 × *g* for 10 minutes, 35 µL of supernatant were transferred to LC vials and inserted into the LC-MS system consisting of a Nexera X2 UHPLC system with two UHPLC pumps (Shimadzu, ’s Hertogenbosch, The Netherlands) connected to a TSQ Quantum Ultra (Thermo Fisher Scientific, Breda, The Netherlands). Separation of 15a was accomplished in a Gemini 3 µm C18 reversed phase HPLC column (50 × 4.6 mm) (Phenomenex, Amstelveen, The Netherlands) using an injection volume of 10 µL and a flow rate of 0.6 ml/min at 40 °C. After elution of 15a with a 9:11 (v/v) ratio of acetonitrile in water with 0.1% TFA, the column was flushed with a gradient up to 90% acetonitrile with 0.1% TFA. The column was re-equilibrated with a 9:11 (v/v) ratio of acetonitrile in water with 0.1% TFA before the next injection. Samples were analyzed by mass spectrometry using Electro Spray Ionization at a voltage of 3000 V. 15a and the internal standard 15b showed the same fragmentation pattern and were quantified by monitoring the transition of 549.17 to 202.08 (*m/z*). The collision energy used was 26 V at a skimmer offset of 16 V, the scan peak width was 0.5 *m/z*, while the scan time was 0.3 seconds. Sheath gas and auxiliary gas pressure (Nitrogen) were set to 60 and 5 respectively (arbitrary units), and the collision gas Argon was set at 1.0 atm.

### Histology and morphometry

Carotid artery cryosections of 5 µm thick were prepared, which were stained with hematoxylin-eosin to determine plaque size and necrotic core size. Morphometric analysis was performed on sections throughout the atherosclerotic lesion (100 µm apart) and at the site of maximal stenosis using Leica Qwin image analysis software. The relative necrotic area was defined as the a-cellular, debris-rich plaque area as percentage of total plaque area, and also quantified using Leica QWin software.

The hearts were dissected just below the atria and sectioned perpendicular to the axis of the aorta, starting within the heart and working in the direction of the aortic arch. Once the aortic root was identified by the appearance of aortic valve leaflets, 10 µm sections were collected. Mean lesion area (in µm^2^) of the aortic root was calculated from six Oil-Red-O stained sections in distal direction starting at the point where all three aortic valve leaflets first appeared.

Macrophage content of the lesions in carotid arteries and aortic roots was stained using a rat monoclonal MOMA2 antibody (1:1000, Serotec, Kidlington, Oxford, UK). Macrophage positive areas were analyzed using computer-assisted colour-gated analysis, and related to the total intimal surface area (Leica QWin). Liver cryosections of 10 µm thick were prepared and subsequently stained with hematoxylin-eosin to analyze liver morphology, which was assessed using a Leica DMRE microscope (Leica Ltd., Cambridge, England).

### RNA isolation and gene expression analysis

Total RNA was extracted from homogenized liver tissue with the guanidium thiocyanate-phenol-bromochloropropane extraction method^38^. RNA concentration, purity and integrity were examined by nanodrop (Nanodrop^®^ Technologies). RNA was reverse transcribed by M-MuLV reverse transcriptase (RevertAid, MBI Fermentas, Landsmeer, The Netherlands) and used for quantitative analysis of the mouse genes ALT, AST, apoH and GC with an ABI PRISM 7700 Taqman apparatus (Applied Biosystems). For qPCR primer pairs refer to Supplementary Table S1.

### Data analysis

All radioligand binding assays were analyzed using Prism 6.0 (GraphPad software, San Diego, CA, USA). All data shown represent means ± SEM of at least three independent experiments performed in duplicate. K_i_ values were calculated from IC_50_ values obtained by non-linear regression analysis of the displacement assays and by using the Cheng-Prusoff equation^47^. Dissociation rate constants *k*_*off*_ were determined by fitting the dissociation data using an exponential decay model. Association rate constants *k*_*on*_ were obtained by converting the apparent association rate constants *k*_*obs*_ according to the equation *k*_*on*_ = (*k*_*obs*_ − *k*_*off*_)/[L], in which L is the radioligand concentration used for the experiments, *k*_*obs*_ is the constant rate determined using exponential association analysis and *k*_*off*_ represents the mean *k*_*off*_ value obtained independently in the dissociation experiments. The kinetic profile of compound 15a was determined by calculation of its kinetic rate index (KRI) value^48^. The KRI values were calculated according to the equation KRI = B_t1_/B_t2_, in which B_t_ represents the specific radioligand binding measured at t_1_ = 15 min and t_2_ = 210 min. In addition, the association and dissociation rates of 15a were obtained by nonlinear regression analysis of the competition association data using the equations described by Motulsky and Mahan^27^. The association and dissociation rates obtained with this model were used to calculate the kinetic *K*_*D*_, according to the equation *K*_*D*_ = *k*_*off*_/*k*_*on*_. In addition, the RT was calculated by obtaining the reciprocal of the *k*_*off*_. Data obtained from *in vivo* experiments are expressed as mean ± SEM. An unpaired two-tailed Student’s t-test was used to compare individual groups. Non-Gaussian distributed data were analyzed using a two-tailed Mann-Whitney U test. A level of P < 0.05 was considered significant.

### Pharmacokinetics-based prediction of target occupancy

The equilibrium target occupancy was calculated by assuming that binding was fast and the drug-target residence time did not prolong the target occupancy. The target occupancy was calculated for each measurement of the drug concentration, according to equation (1), in which [*C*] is the concentration of the drug in the blood and K_D_ is the drug target affinity.

1 $$Target\,Occupancy( \% )=\frac{[C]}{[C]+{K}_{D}}\times 100 \% $$

In addition, a simple mathematical model was used to simulate target occupancy for the observed *in vivo* pharmacokinetics and *in vitro* binding kinetics according to the equations in the next section (equations (3–5)).

To determine the maximal value of *k*_*off*_ that would prolong the receptor occupancy even further, we used equation (2)^28^, in which BF represents the percentage of target occupancy divided by 100 and *t*_1/2*el*_ represents the plasma elimination half-life (0.693/*k*_*el*_). In this case we set BF to 0.90, as 90% represents the minimum value of target occupancy to achieve efficacy. To obtain the elimination rate constant of compound 15a, a linear regression analysis of the natural logarithm of the concentrations versus time was performed, and the slope coefficient was used as the elimination rate constant *k*_*el*_.

2 $${k}_{off} < 0.693\cdot (1-BF)/{t}_{1/2el}$$

### Differential equations for the simulations of target occupancy

The concentrations of unbound drug in the blood [*C*] and target-bound drug [*LR*] were modeled according to equations (3–5). In these equations, *k*_*el*_ is the first-order elimination rate constant, *k*_*on*_ is the second-order association rate constant, *k*_*off*_ is the first-order dissociation rate constant, [*R*_*free*_] is the concentration of unbound receptor and [*R*_*tot*_] is the concentration of bound plus unbound receptor, which is assumed to be constant.

3 $$\frac{d[C]}{dt}=-{k}_{el}\cdot [C]-{k}_{on}\cdot [C]\cdot [{R}_{free}]+{k}_{off}\cdot [LR]$$

4 $$[{R}_{free}]=[{R}_{tot}]-[LR]$$

The concentration of the target-bound drug is calculated according to equation (5):

5 $$\frac{d[LR]}{dt}={k}_{on}\cdot [C]\cdot [{R}_{free}]-{k}_{off}\cdot [LR]$$

Parameter values: for the simulations in Fig. 8b, *k*_*el*_ was set to 0.0647 hr^−1^, based on the slope of the linear regression of the pharmacokinetic data (Fig. 8a). On basis of in-house measurements, *k*_*off*_ was set to 3.06 hr^−1^ (corresponding to 0.051 min^−1^, as shown in Table 1) and the *K*_*D*_ was set to 10 nM, which corresponds to the K_i_ value of 15a determined in [^3^H]INCB3344 displacement assays. The K_i_ value was used instead of the *K*_*D*_ (from Table 1) as this represents a more conservative choice with regard to target saturation. The receptor concentration was set to an arbitrarily low number of 1 pM.

## Electronic supplementary material

Supplementary Information
