# Supplementary material for: A novel CCR2 antagonist inhibits atherogenesis in apoE deficient mice by achieving high receptor occupancy
Source: Sci Rep. 2017 Mar 3;7:52. doi: 10.1038/s41598-017-00104-z (PMC5427923; doi:10.1038/s41598-017-00104-z)
Supplement: Supplementary file 1 — Supplementary Information [file 41598_2017_104_MOESM1_ESM.pdf]

Supplementary Information

A novel CCR2 antagonist inhibits atherogenesis in apoE deficient mice by achieving high receptor occupancy

Ilze Bot[1,+], Natalia V. Ortiz Zacarías[2,+], Wilhelmus E.A. de Witte[3], Henk de Vries[2], Peter J. van Santbrink[1], Daniël van der Velden[1], Mara J. Kröner[1], Dirk-Jan van der Berg[3], Dean Stamos[4], Elizabeth C.M. de Lange[3], Johan Kuiper[1], Adriaan P. IJzerman[2], Laura H. Heitman[2,]*

[1]Division of Biopharmaceutics, [2]Division of Medicinal Chemistry and [3]Division of Pharmacology, Leiden Academic Centre for Drug Research (LACDR), Leiden University, Leiden, the Netherlands.

[3]Vertex Pharmaceuticals, San Diego, CA, USA.

*l.h.heitman@lacdr.leidenuniv.nl

[+]these authors contributed equally to this work

Gene	***Forward primer (5’-3’)***	***Reverse primer (3’-5’)***
ALT	CCCTGACATGTTCTTCTGTCTGTGCC	CCTCAGTTTCTCCAGCAGCACCC
AST	TGACCGGATTCTGACCATGAGATCCG	GGTCAAGCCGCACATGTTGATCCG
apoH	CATGAGACATACAAGCTGGACGGCC	TACAGCACGGTGGCTTTCTTAACGG
GC	GCAGAACGGCTAAGGACAAAA	AGTCCGAGTGTTTCTCCACCAT
Rpl27	CGCCAAGCGATCCAAGATCAAGTCC	AGCTGGGTCCCTGAACACATCCTTG
β-actin	AACCGTGAAAAGATGACCCAGAT	CACAGCCTGGATGGCTACGTA
TAF7	AGTCTGGGCATGTCAACCTGAA	CGTAACACAAGGCAAATCGACCA

Supplementary Table S1. qPCR gene primer sequences. All gene expression analysis was performed using three housekeeping genes (Rpl27, β-actin and TAF7). Abbreviations: ALT: alanine aminotransferase; AST: aspartate aminotransferase; apoH: apolipoprotein H; GC: group specific component; Rpl27: 60s ribosomal protein ligand 27; TAF7: TATA-box-binding protein.

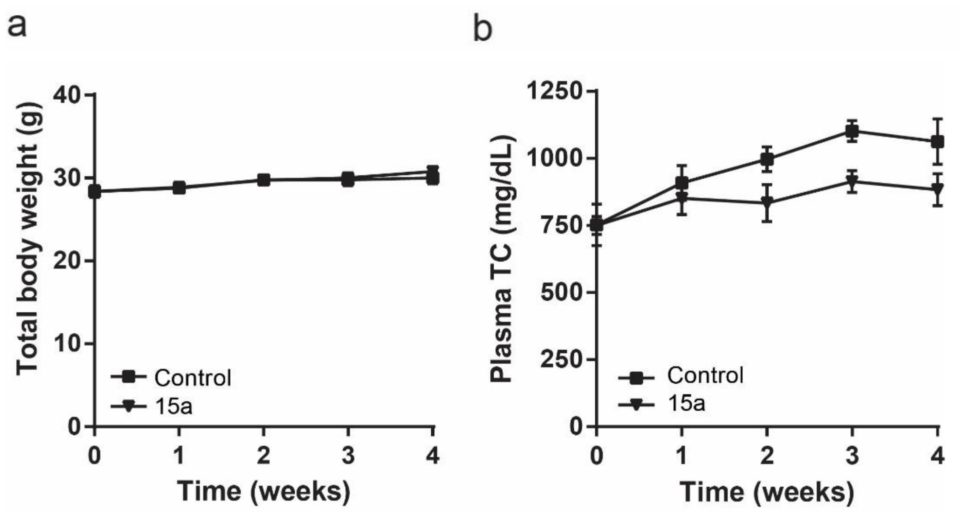

Supplementary Figure S1. Effect of 15a on body weight and cholesterol levels. Total body weight (**a**) and plasma total cholesterol (TC) levels (**b**) were not significantly affected by treatment with 15a. Graphs shown are representative from one experiment with n=10 controls versus n=9 15a-treated mice.

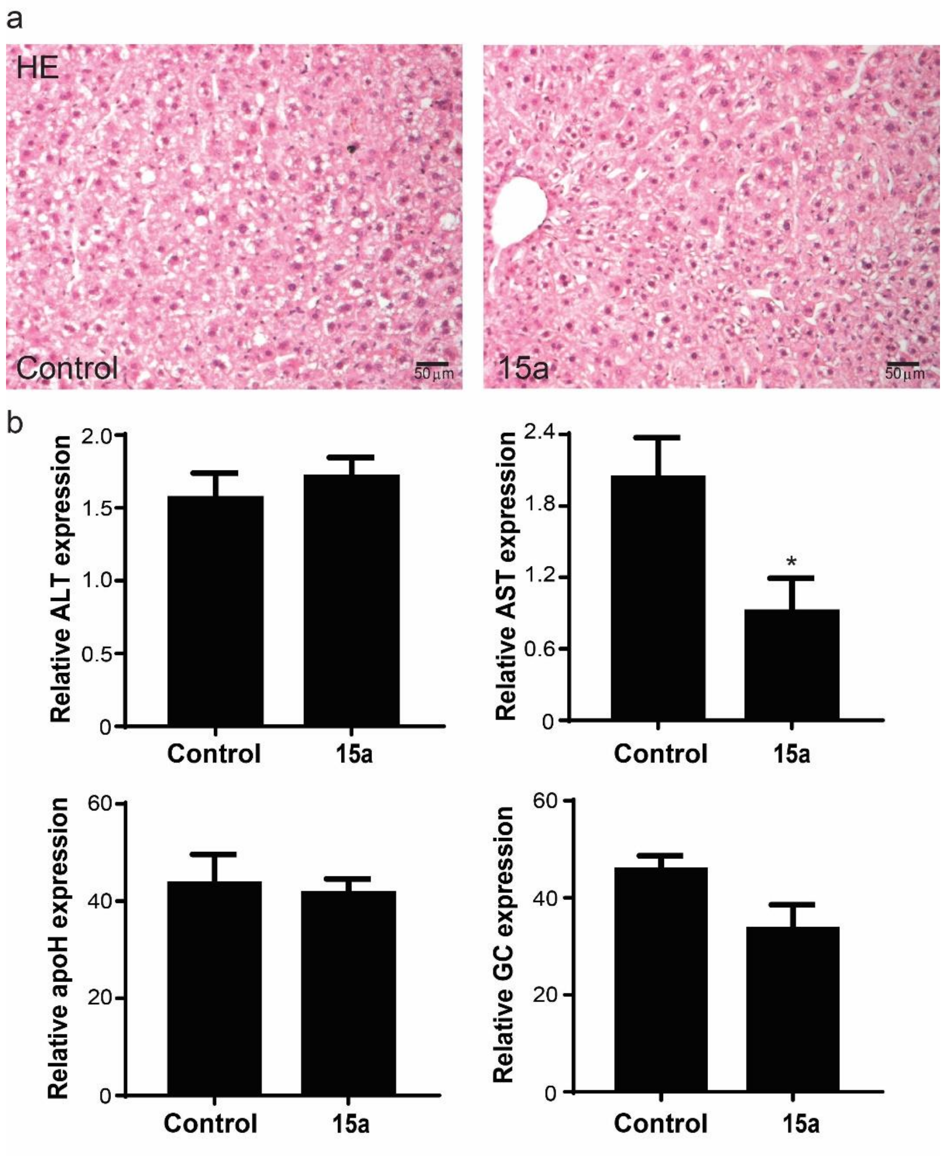

Supplementary Figure S2. 15a does not affect liver function. (**a**) Histological analysis of the liver did not reveal any adverse effects of the 15a treatment as shown by an HE staining. (**b**) Also, gene expression analysis did not show any differences in mRNA expression of liver toxicity markers ALT, apoH and GC, while liver AST expression levels were even somewhat decreased (*P=0.02). Graphs shown are representative from one experiment with n=6 per group.

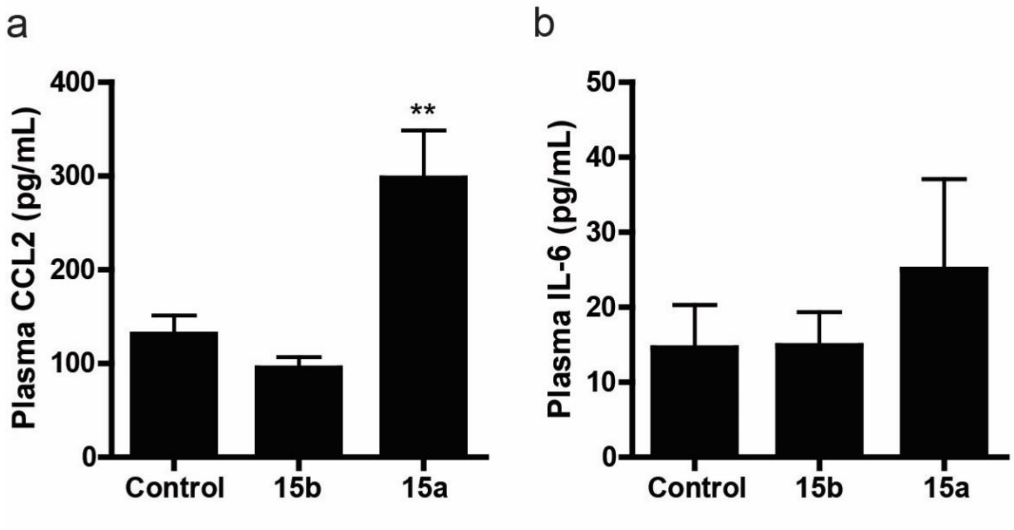

Supplementary Figure S3. Effect of 15a on CCL2 and IL-6 plasma levels. Plasma CCL2 levels were increased upon treatment with 15a (**a**), while IL-6 levels remained unaffected by treatment with 15a (**b**). **P<0.01. Graphs shown are representative from one experiment with n=10 controls versus n=9 15a-treated mice.

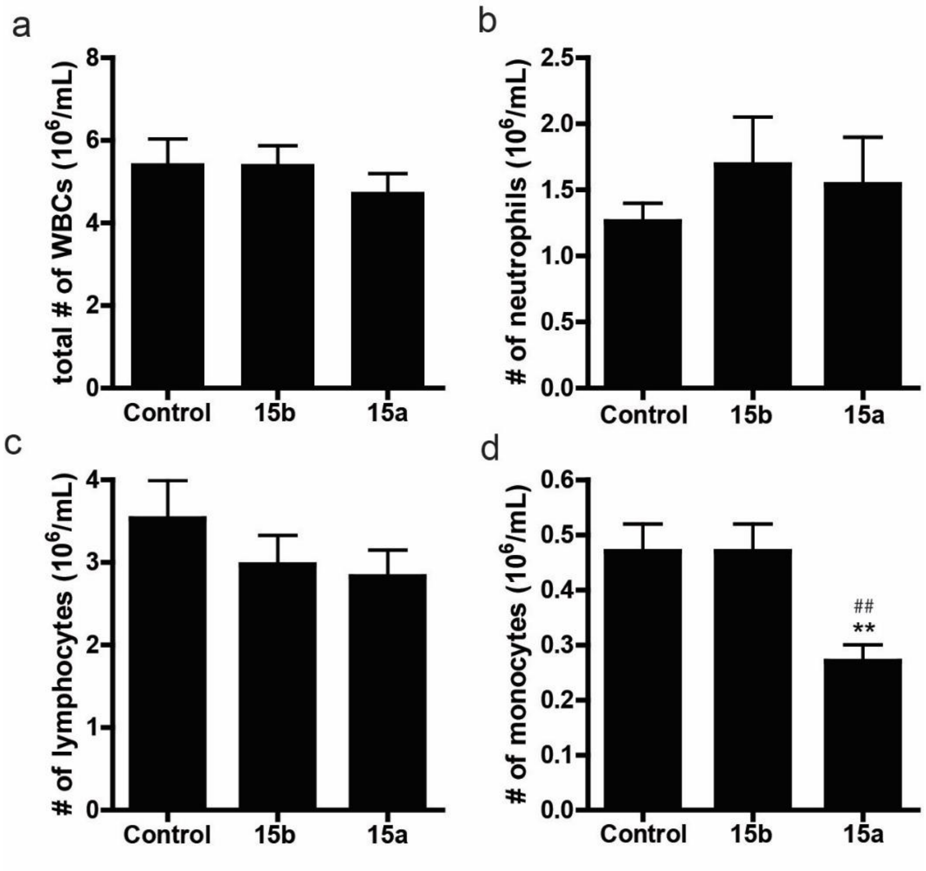

Supplementary Figure S4. Effect of 15a on white blood cells. No differences were found in circulating total white blood cell (WBCs) (**a**), neutrophil (**b**) or lymphocyte (**c**) counts between the groups, but 15a significantly reduced the number of circulating monocytes (**d**). **P<0.01. Graphs shown are representative from one experiment with n=10 controls versus n=9 15a-treated mice.

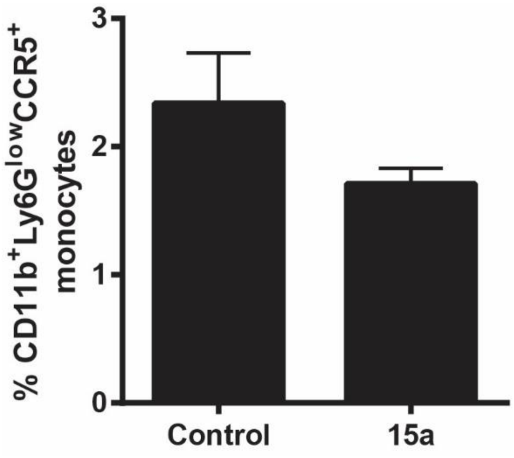

Supplementary Figure S5. Effect of 15a on CCR5$^+$ monocytes. Treatment with 15a did not affect the circulating % of CD11b$^+$Ly6G^{low}CCR5$^+$ monocytes. Graphs shown are representative from one experiment with n=6 per group.
